# Managing dental emergencies: A descriptive study of the effects of a multimodal educational intervention for primary care providers at six months

**DOI:** 10.1186/1472-6920-12-103

**Published:** 2012-10-30

**Authors:** Tony Skapetis, Tania Gerzina, Wendy Hu

**Affiliations:** 1 Clinical Director of Education Westmead Centre for Oral Health, Western Sydney Local Health District, University of Sydney faculty of Dentistry, PO Box 533, Wentworthville, NSW 2145, Australia; 2 Jaw Function and Orofacial Pain Unit, Faculty of Dentistry, Division of Health, Westmead Centre for Oral Health, University of Sydney, Westmead Australia 2145, Australia; 3 University of Western Sydney School of Medicine, Locked Bag 1797, Penrith, NSW 2751, Australia

**Keywords:** Dental, Emergencies, Education, Medical, Proficiency, Confidence, Model

## Abstract

**Background:**

Clinicians providing primary emergency medical care often receive little training in the management of dental emergencies. A multimodal educational intervention was designed to address this lack of training. Sustained competency in managing dental emergencies and thus the confidence to provide this care well after an educational intervention is of particular importance for remote and rural healthcare providers where access to professional development training may be lacking.

**Methods:**

A descriptive study design with a survey instrument was used to evaluate the effectiveness of a brief educational intervention for primary care clinicians. The survey was offered immediately before and at six months following the intervention. A Wilcoxon signed rank test was performed on pre and six month post-workshop matched pair responses, measuring self-reported proficiency in managing dental emergencies. The level of significance was set at p < 0.001. Confidence intervals (CI) were calculated for participants who scored an improved proficiency.

**Results:**

The educational intervention was associated with a significant and sustained increase in proficiency and confidence to treat, especially in oral local anaesthesia, management of avulsed teeth and dental trauma, as reported by clinicians at six months after the education. This was associated with a greater number of cases where dental local anaesthesia was utilised by the participants. Comments from participants before the intervention, noted the lack of dental topics in professional training.

**Conclusions:**

The sustained effects of a brief multimodal educational intervention in managing dental emergencies on practice confidence and proficiency demonstrates its value as an educational model that could be applied to other settings and health professional groups providing emergency primary care, particularly in rural and remote settings.

## Background

Dental emergencies commonly present to medical personnel rather than to dental surgeries due to ease of access, including location, convenience and affordability [1]. Reported barriers to accessing dental services include practice hours and lack of insurance [2]. Common emergency presentations include dental pain, bleeding, infection and trauma [3]. In the US, for example, dental complaints have been reported to represent 1.3% of US Emergency Department (ED) visits between 1997 and 2000, or 738,000 visits annually [4]. In another review, oral/dental complaints made up 0.3% of all consultations across 30 general medical practices [5]. Often such dental emergencies require simple but specific management to prevent complex post-emergency sequelae for the patient. These sequelae can include prolonged discomfort, premature tooth loss, or need for major restorative care to restore oral function. Despite the frequency of dental emergency presentations, medical personnel are reported as lacking both knowledge and training in the management of such presentations [6]. Appropriately targeted medical education can be effective in changing physician performance when utilising multiple media and instructional techniques [7].

The purpose of this study was to test whether a well designed interactive and multimodal educational intervention on the management of dental emergencies would have a sustained effect on self-reported proficiency, and on the number of such cases treated by primary care providers over a six month period.

## Methods

The study group consisted of 242 workshop participants (Table 1) that included physicians, nurse practitioners and medical students. All participants completed an identical four hour workshop, as the core educational intervention, on managing common dental emergencies. This was delivered by the same presenter across three Australian states in 11 hospital Emergency Departments (ED) and rural medical conferences held by the peak professional associations for rural physicians in Australia. The choice of site was by open invitation from ED directors and conference organisers, with participants volunteering attendance. All clinicians working in the host EDs or conferences at the time of the workshops were eligible to participate in the study and volunteered consent to be surveyed before the workshop (T1) and six months later (T2).

**Table 1:** Professional background of participants in educational intervention

	**Number**	**% respondents**
Specialist ED physician	18	7.4
GP	62	25.6
Emergency registrar	70	28.9
Non-emergency registrar	24	9.9
Intern/Resident	27	11.2
Career medical officer	26	10.7
Nurse Practitioner	9	3.7
Student	6	2.5
Total	242	100.0

**Figure 1:**
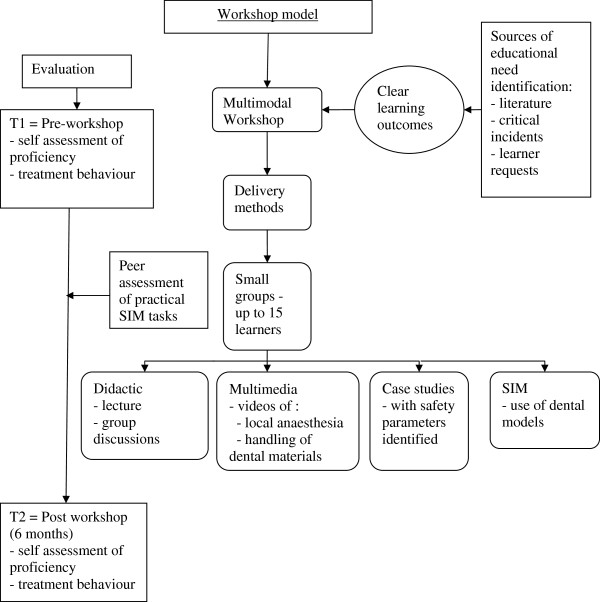
Workshop Model.

The workshop (Figure 1) comprised didactic, multimedia (video), case study, simulation (SIM) teaching, peer assessment and self assessment components. Videos of dental local anaesthesia, manipulation of dental materials and the splinting of teeth, were employed. The SIM task involved a practical demonstration using plastic dental models of a complicated crown fracture repair as well as splinting of teeth following avulsion with re-implantation, using Glass Ionomer Cement (a dental restorative material). Participants were then asked to perform these tasks while being assessed by fellow workshop participants using a criterion checklist. Similarly, following instruction, participants were asked to demonstrate through SIM (using a torch and long cotton tip instead of needle and syringe), administration of dental anaesthesia on their peers whilst being assisted and assessed by the educator.

**Figure 2:**
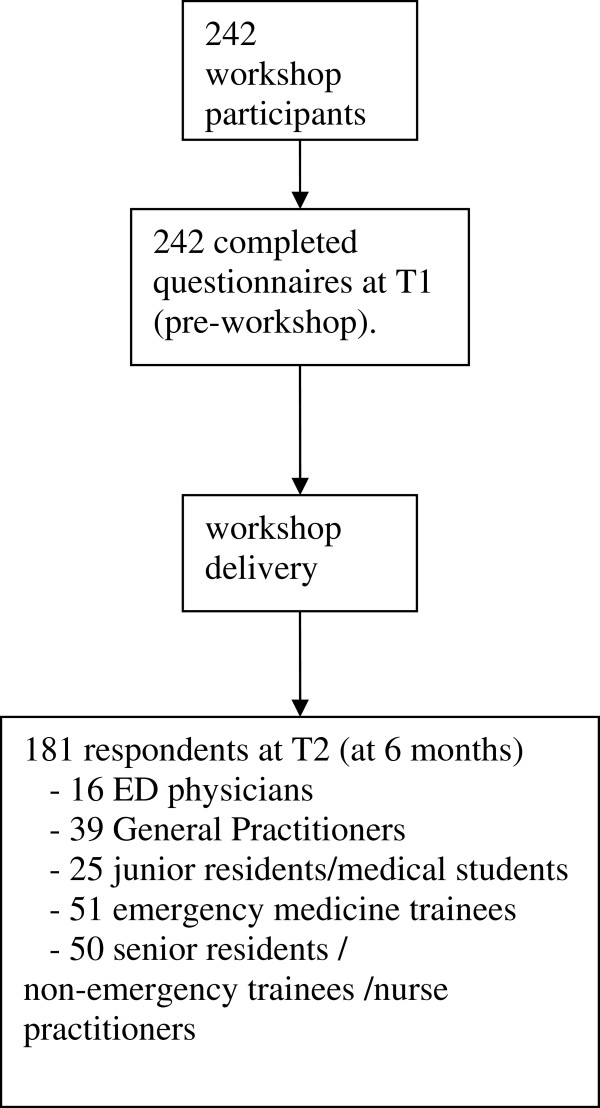
Sequence of survey completion for participants.

The need for this type of training had been identified by requests for training in dental emergencies and a review of the literature [8], and confirmed by presentations of poorly managed dental emergencies to the author (TS) in a large dental hospital emergency department. All participants completed a pre-workshop questionnaire ( Additional file 1 and reported here as “T1”), and were invited to complete a follow up questionnaire (T2), which included two additional questions. The first asked if there had been any further education and the second for perceived proficiency in the treatment of dental emergency presentations within the six month follow up period. The follow up survey was distributed through email and post (Figure 2).

Open ended comments were also invited at time points T1 and T2. This study was approved by Concord Repatriation General Hospital Human Research Ethics Committee (CH62/6/2009-010).

Data was de-identified and paired analysis performed using Wilcoxon signed rank test and frequencies calculated using SPSS® version 15. Confidence intervals (CI) were calculated for participants who scored an improved proficiency.

The 13 item survey instrument was developed by the authors, reviewed by a multidisciplinary professional panel for content validity including two dental and medical academics, emergency physicians, general practitioners and one registered nurse. Minor language changes were subsequently made. It was piloted amongst a group of ED clinicians including two nurse practitioners and sixteen physicians following which wording modifications were further made to improve construct validity. Questionnaires were further tested for reliability using Cronbach’s Split Half reliability analysis (0.79) and for discriminant validity through the use of a “dummy” question which asked for a response surrounding a topic not covered by the workshop content.

## Results

### Characteristics of respondents at six month follow up

The response rate at 6 month T2 (post-workshop) follow up was 74.8% (181/242 workshop participants). Of the 181 respondents, 60% were male, 57% had graduated up to 10 years prior to the workshop, and 53.6% were practicing in rural, 37% in urban and 9.4% in remote locations at the time of the survey. There was no significant association observed between practice location and the type of dental emergencies treated.

In comparing the demographic characteristics of T2 respondents and non-respondents using data from the T1 (pre workshop) questionnaire, there were statistically significant differences regarding respondents’ professional background, previous experience of dental education, provision of ED services and the number of dental trauma cases treated (chi-square test p < 0.05). Specialist ED physicians (89%) were most likely to respond while general practitioners (GP) were least likely to be respondents. Study participants having received previous dental education (83%), those providing ED services (77%) and those that had treated one or more cases of dental trauma (83%) in the preceding 12 months, were more likely to be T2 respondents.

Of the T2 respondents, 96.6% had not received any further dental education in the 6 months following the workshop.

### Number and type of dental emergencies treated before the workshop and at 6 month follow up

**Figure 3:**
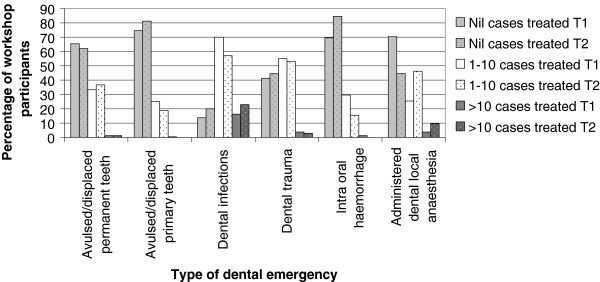
Annual rates of dental emergencies treated prior to workshop at T1 and following workshop at T2 with dotted columns representing rates for the same procedure at T2.

Annual rates of dental emergencies participants reported treating prior to the workshop (T1) and in the 6 months following the workshop (T2) are summarised in Figure 3.

A comparison of the number and type of dental emergencies treated by T2 respondents before and after the workshop (paired Wilcoxon P < 0.001) showed significant increase in the number of cases treated involving dental anaesthesia and a decrease in cases involving intra-oral haemorrhage. However, 80.5% of T2 respondents had not treated any dental emergencies in the 6 months since the workshop; citing reasons such as a lack of presenting patients (81%), lack of confidence and/or insufficient dental equipment (9.8%).

When asked if there were any type of dental emergencies for which T2 respondents felt not proficient in providing emergency treatment, 15.6% cited cases including multiple complex tooth injuries, jaw fractures, anaesthesia difficulties and paediatric presentations. Open ended questions inviting comments at T1 (n = 28), largely acknowledged the lack of previous education surrounding the management of dental emergencies.

### Self reported change in proficiency at 6 months after the workshop

Self reported change in proficiency between T1 and T2 is summarised in Table 2 with significance set at p <0.001 using paired Wilcoxon statistical analysis.

**Table 2:** Self-Reported change in proficiency at 6 month follow up (T2 compared to T1)

	**% worse**	**% unchanged**	**% improved (CI)**	**p-value**
proficient in describing a dental emergency to a Dentist	1.2	21.6	77.2 (71-83)	<0.001
able to assess the urgency of a dental emergency presenting to ED	1.1	32.2	66.7 (60-74)	<0.001
able to give appropriate dental local anaesthesia		17.8	82.2 (77-88)	<0.001
able to control haemorrhage from inside the mouth	6.1	37.3	56.6 (46-64)	<0.001
able to place sutures correctly inside the mouth	3.9	42.3	53.8 (47-61)	<0.001
Can provide appropriate emergency treatment and management for:				
avulsed or displaced permanent tooth	1.2	22.7	76.1 (70-82)	<0.001
avulsed or displaced primary tooth	1.7	20.6	77.7 (72-84)	<0.001
a dental infection	5.0	36.1	58.9 (52-66)	<0.001
dental trauma	2.3	18.3	79.4 (74-85)	<0.001
pericoronitis*	4.4	39.4	56.2 (49-63)	<0.001

* “Dummy” question included to support construct reliability.

## Discussion

### Educational theory

Andragogy is often defined as the science of teaching adults. This concept is often idealised yet rarely embodied when developing workshops for medical care providers. We aimed to design a multimodal workshop which embodied adult learning strategies including SIM, case studies and self evaluation as suggested by Knowles [9]. Additionally, this education considered the needs and wants of the target learners (Figure 3) which is consistent with Tylers model [10] and experiential learning proponents [11]. The workshop attempted to diversify the range of treatment options offered in an area not generally managed by primary care providers, a process which has been called ‘productive diversity’ and ‘new learning [12]. These authors describe how new learning is characterised by transformative education facilitating behavioural (practice) change. This is highlighted by one of the participants in their open comments at T2.

The majority of other comments (n = 103) were positive, relating to the relevance, content and delivery of the education.

### Workshop elements

The workshop was supported by learning outcomes specific to each topic presented (Table 3). Underpinning the workshop was a didactic presentation using photographs of clinical cases, supporting literature and colour handouts to enhance the learning experience. The topics of tooth nomenclature and traumatic dental injuries(TDI) included instruction on the FDI (World Dental Federation) [13] and WHO (World Health organisation) [14] classifications respectively. The local anaesthesia component included instruction on dental infiltration and block anaesthesia and the TDI component included instruction on the management of crown fractures, luxation and avulsion injuries. Management of intra-oral haemorrhage included instruction on suturing methods and management of dental infections included education on origin and spread of odontogenic infections and antibiotic choices.

**Table 3:** Workshop topic learning outcomes

**Topic**	**Learning outcomes**
Dental Nomenclature	- Understanding the main components of tooth anatomy and structure.
	- The ability to describe and record both the primary and secondary dentition using appropriate nomenclature.
Dental Anaesthesia	- The ability to manage acute dental pain effectively.
	- The ability to give infiltration & block anaesthesia around the mouth.
Dental Trauma	**-** Ability to provide effective emergency treatment for common dental trauma.
Intra-oral haemorrhage	**-** Ability to provide effective emergency treatment for intra-oral haemorrhage.
Dental Infections	- Effective first line management of commonly occurring dental infections.

Similar educational interventions combining didactic with other instructional techniques have been documented [15-18]. This study differs with regards to subject matter and context, with other workshop style educational interventions in the management of dental emergencies having not previously been reported from Australia. The variety of teaching techniques together with assessment methods reinforced the delivery of workshop learning outcomes.

### Survey respondents

Primary care providers surveyed included physicians, nurse practitioners and students. The proportion of participants in each group reflected staffing levels commonly found in Australian EDs, where most of the workshops were conducted. The role of the medical student is generally limited to treatment of dental emergencies under supervision with physicians and nurse practitioners (who are capable of prescribing and administering local anaesthesia) able to provide independent treatment within their scope of training [19].

Respondents at T2 were more likely to be ED physicians, have had previous dental education, be providing ED services and treating more dental trauma.

Proficiency when delivering dental local anaesthesia showed the greatest improvement and this skill was reported as the most utilised during the 6 month follow up period. Self-reported proficiency was sustained during the 6 month follow up period.

Both T1 and T2 questionnaires asked participants to make an evaluation of their own proficiency in managing common dental emergencies. Self-assessment is an important part of self-awareness and an essential component of critical thinking. It is also a common focus in health professional curricula. Self assessment has been reported useful in improving learning [20] by providing insights into identifying learning needs, although it can be inaccurate [21].

In the pre-workshop and follow up questionnaires (T1,T2), professional category of respondents was statistically different from those of non-respondents. ED physicians were found to be most likely to respond to the 6 month follow up questionnaire (T2) and general practitioners were the least likely. This may be due to ED physicians placing a greater importance on this topic as they may be more likely to encounter dental emergencies. Respondents were also more likely to be currently delivering ED services (77.1% versus 47.4% for participants not providing ED services) and have treated more cases of dental trauma.

These statistically significant differences between respondents and non-respondents to the follow up questionnaire may suggest an element of responder bias and this should be considered when translating our results to other settings and professional groups.

Nevertheless, the high response rate to the 6 month follow up questionnaire as well as differences between respondents and non-respondents highlights the importance of topic relevancy in educational interventions and therefore its effectiveness in promoting learning. Relevancy of training has been reported to improve learning outcomes in other literature [22].

A majority of T2 respondents (84.4%) reported that they had not encountered any dental emergencies which they were not proficient in managing. This would suggest that the topics covered by the workshop, including dental nomenclature, dental trauma, intra-oral haemorrhage control, dental infections and dental anaesthesia, adequately covered the majority of common presentations facing clinicians, indicating good specificity.

### Dental emergencies treated

With regards to the treatment of intra-oral haemorrhage and performance of dental anaesthesia, results shown in Figures 3 demonstrated a significant decrease between T1 and T2 in the number of cases treated per annum. More respondents had either not treated any intra-oral haemorrhage cases (84.4%) or had treated 1-10 cases (15.6%) after the workshop (T2), while at T1, these percentages were 69.4% and 29.4% respectively. This result is heavily influenced by the types of cases presenting at different times and is consistent with the smaller proficiency gain (56.6%) surrounding this topic. Conversely, at T2, more dental local anaesthesia was utilised with less than half of respondents having treated “no such cases” (44.4%) and almost half (46.1%) having treated 1-10 cases. This outcome demonstrates a significant change which suggests a behaviour shift towards using local anaesthesia more frequently as a result of the workshop education. The study was not designed to measure the quality of such treatment. There was also a significant association between T2 respondents who had not treated any cases of permanent tooth avulsion/displacement. The reported absence of previous dental education prior to the workshop and during the six months, suggests that the workshop would enhance knowledge and skills.

### Proficiency change and sustainability

The majority of T2 respondents (96.6%) had not received any further dental education during the follow up period so any self reported change in proficiency would not be due to other training. Table 2 results show that there were sustained improvements in self-reported proficiency in most of the topics covered by the workshop at T2. Topics showing the least improvement as well as an element of negative improvement included: placing sutures inside the mouth, management of pericoronitis (the “dummy” question) and dental infections as well as controlling intra-oral haemorrhage. This may be explained in several ways; firstly, the participants may already have pre-existing proficiency in this topic prior to the workshop, the workshop may not have been effective, participants may not have had opportunities to practice skills that they had learnt at the workshop or the survey tool failed to capture differences, for example due to limited recall. In addition, the small improvement reported for management of pericoronitis was anticipated as this was the dummy question concerning a topic that was not covered by the workshop but was included to test survey reliability [23,24]. It is possible that respondents actually improved their own understanding on this topic by themselves. The modest improvement for placing sutures and controlling intra-oral haemorrhage could be attributed to lower numbers of such presentations during the post workshop period (Figure 3) and thus opportunities to practice newly acquired skills. Conversely, the modest improvement seen in the management of dental infection may be due to pre-existing proficiency as such presentations are common in medical practice [25].

### Limitations and strengths

This study is limited by its reliance on respondent recall at the 6 month follow up of treated cases. However, this time period was chosen as a balance between testing whether effects of the workshop were sustained, and the time period when recall is expected to be reasonably accurate. Literature has generally reported a decline in retention and recall of clinical information at three months following an educational intervention amongst medical students of between 10 and 19% [26] – our follow up period was double this and was able to demonstrate sustained increases from time zero.

There may also be elements of response bias given the differences between respondents and non-respondents at T2, although our detailed analysis of the differences will allow better transferability of the results to other settings and professional groups. Further bias may have been introduced through the Hawthorne effect as participants were aware that they would be followed up after six months. However, the size of the proficiency gains together with the many positive comments from the open ended question, assisted to triangulate the results as suggested by Holden [27] and quantify this effect. A further weakness relates to the study’s reliance on self assessment as the main form of evaluation, although peer assessment using a criterion checklist was also employed to enhance the learning experiences. The checklist related to simulated repair of a dental avulsion using dental models and described key features of the task subunits to aid the peer assessment. Such checklists have been used as aids in peer assessment of competence [28].

Strengths of this study include the sample size, that significant effects were shown with the workshop being conducted at multiple sites and with participants with diverse professional backgrounds. This demonstrates that the intervention was effective in different settings and with different health professional groups and levels of expertise. The high six month response rate and the link between the educational intervention and respondents’ performance of the skills suggests that learning was applied in real clinical settings during the follow up period. These strengths suggest that the educational model for this workshop will be effective in other settings and with different health professional participants.

Suggestions for further research in this area may include a more accurate measure of the number of dental emergencies treated for example through a medical record audit over a longer period, a measure of the quality of the treatment provided and the most effective format and timing of educational reinforcement following workshops.

## Conclusions

A model for a single multimodal educational intervention in management of dental emergencies that demonstrates a significant increase in the use of dental local anaesthesia by emergency care clinicians and improved self reported proficiency is presented. Self reported proficiency was most improved in administering dental local anaesthesia for managing dental presentations at six month follow up. Clinicians’ self reported improvement in proficiency was sustained beyond the workshop for at least six months. This may be due to the use of multiple instructional strategies and topic relevancy. Our findings suggest that the educational model used for this intervention will be effective in other settings with a range of health professionals who are called upon to treat dental emergency presentations.

## Abbreviations

ED: Emergency department; T1: Time 1 – pre-workshop; T2: Time 2 – six months post-workshop; GP: General practitioner; SIM: Simulation; TDI: Traumatic dental injuries.

## Competing interests

The authors declare that there are no financial or non-financial competing interests in relation to this manuscript.

## Authors’ contributions

*TS* - principal author and researcher for this study. *TG* - contributed to design, early drafts, editing and final approval of this paper. *WH* - contributed to design, early drafts, editing and final approval of this paper. All authors read and approved the final manuscript.

## Authors’ information

Principal author: *Dr Tony Skapetis*

- BDS, Med (Adult Education).

- Clinical Director of Education, Westmead Centre for Oral Health, Western Sydney Local Health District. Clinical Senior Lecturer, Faculty of Dentistry, University of Sydney

Co-authors: *Associate Professor Tania Gerzina*

- Associate Professor Dental Education, Faculty of Dentistry University of Sydney,

Co-authors: *Professor Wendy Hu*

- Professor of Medical Education, School of Medicine, University of Western Sydney.

## Pre-publication history

The pre-publication history for this paper can be accessed here:

http://www.biomedcentral.com/1472-6920/12/103/prepub

## Supplementary Material

Additional file 1 **Pre workshop questionnaire.** Questionnaire type survey instrument completed by participants prior to workshop (T1). Click here for file
