# Supplementary material for: Managing dental emergencies: A descriptive study of the effects of a multimodal educational intervention for primary care providers at six months
Source: BMC Med Educ. 2012 Oct 30;12:103. doi: 10.1186/1472-6920-12-103 (PMC3534540; doi:10.1186/1472-6920-12-103)
Supplement: Additional file 1 — Pre workshop questionnaire. Questionnaire type survey instrument completed by participants prior to workshop (T1). [file 1472-6920-12-103-S1.pdf]

The University of Sydney
NSW 2006 AUSTRALIA

SYDNEY WEST AREA HEALTH SERVICE | NSW HEALTH

Dr Tony Skapetis
Tony_Skapetis@wsahs.nsw.gov.au
Ph: (02) 98457167

Dental Education for Emergency Medical Teams

ID Number: ________________

Dental Emergency Pre Workshop Self Rating Questionnaire

This survey will help collection information surrounding the management of dental emergencies by emergency medical staff. Your responses will be kept strictly confidential and will not be used to identify you to others.

1. What is your gender?: Male ☐ Female ☐

2. Years since graduation: ☐ <5 yrs ☐ 5-10yrs ☐ 11-15yrs ☐ >15yrs

3. Please describe yourself: Specialist ☐ GP ☐ RN ☐ Emergency Registrar ☐
   Non–Emergency Registrar ☐ Intern/Resident ☐ Career Medical Officer ☐
   Nurse Practitioner ☐ Student ☐ Other ________________

4 Location of practice: urban ☐ rural ☐ remote ☐

5. Have you received any previous dental education or training? Yes ☐ No ☐
   If answered Yes, what form did it take?
   - Web-based resource/s Yes ☐ No ☐
   - Books and journal article/s Yes ☐ No ☐
   - Lecture/s Yes ☐ No ☐
   - practical workshop/s Yes ☐ No ☐
   - Other ________________

6. Do you provide emergency medical services? Yes ☐ No ☐

7. In the last 12 months have you provided any emergency treatment for the following:
   (circle Yes or No and if answering yes, list number of cases)

		Number of Cases (only if answered Yes)	
Avulsed or displaced permanent tooth	Yes	☐	No
Avulsed or displaced primary tooth	Yes	☐	No
A dental infection	Yes	☐	No
Dental trauma	Yes	☐	No
Intraoral haemorrhage	Yes	☐	No
Dental pain using dental local anaesthesia	Yes	☐	No

Continued overleaf

Please answer the following questions using the scale below and *circle* the most appropriate number:

1 = not proficient 2 = less proficient 3 = proficient 4 = highly proficient

		Not proficient			Highly proficient
8.	Would you feel proficient in describing a dental emergency that presents in ED, to a Dentist?	1	2	3	4
9.	A dental emergency presents to you in ED. Do you think you can assess it's urgency?	1	2	3	4
10.	Do you feel that you are able to give appropriate dental local anaesthesia?	1	2	3	4
11.	Would you be able to control haemorrhage from inside the mouth?	1	2	3	4
12.	Do you feel able to place sutures correctly inside the mouth?	1	2	3	4

13. Do you feel that you can provide appropriate emergency treatment and management for the following?

13.1	An avulsed or displaced permanent tooth	1	2	3	4
13.2	An avulsed or displaced primary tooth	1	2	3	4
13.3	A dental infection	1	2	3	4
13.4	Dental trauma	1	2	3	4
13.5	Pericoronitis	1	2	3	4

Please make any other comments here:
